# Glucosylsphingosine (Lyso-Gb1): An Update on Its Use as a Biomarker in Gaucher Disease

**DOI:** 10.3390/ijms27041705

**Published:** 2026-02-10

**Authors:** Francesca Carubbi, Silvia Linari, Marco Spada

**Affiliations:** 1 Unit of Internal Metabolic Medicine, University of Modena and Reggio Emilia, Baggiovara University Hospital AOU of Modena, 41126 Modena, Italy; 2 Center for Bleeding Disorders and Coagulation, Careggi University Hospital, 50134 Florence, Italy; linaris@aou-careggi.toscana.it; 3 Department of Pediatrics, University of Torino, 10126 Torino, Italy; marco.spada@unito.it

**Keywords:** Gaucher disease, lyso-Gb1, glucosylsphingosine, biomarkers, diagnosis, response to therapy, lysosomal storage diseases

## Abstract

Gaucher disease (GD) is a lysosomal storage disorder caused by mutations in the glucocerebrosidase gene (*GBA1*), leading to acid β-glucosidase deficiency and the accumulation of glucosylceramide-derived glycosphingolipids. Its three phenotypes (non-neuronopathic, acute neuronopathic, and chronic neuronopathic) have variable clinical presentations including hepatosplenomegaly, cytopenia, bone disease, and neurological involvement. Early diagnosis and treatment are critical for improving outcomes, but GD is under-recognized due to non-specific symptoms and limited access to appropriate diagnostic testing. Glucosylsphingosine (lyso-Gb1), a deacylated metabolite of glucosylceramide, has been identified as a candidate biomarker for diagnosis and monitoring. This narrative review examines the role of biomarkers in GD, focusing on lyso-Gb1 as a potential diagnostic and prognostic biomarker. Lyso-Gb1 is markedly elevated in GD patients and correlates with disease burden, severity, and response to therapy. It is detectable in plasma and dried blood spots, making it suitable for newborn screening, diagnosis, and monitoring. Lyso-Gb1 is a sensitive and specific biomarker for GD, facilitating early detection, guiding treatment decisions, and enabling personalized disease management. Lyso-Gb1 levels reflect substrate accumulation and therapeutic response more reliably than other biomarkers such as chitotriosidase or CCL18. Ongoing research aims to refine diagnostic thresholds and integrate lyso-Gb1 monitoring into routine clinical practice for optimal patient outcomes.

## 1. Introduction

Gaucher disease (GD) is the most common lysosomal storage disease. It is a rare, inherited disorder that follows an autosomal recessive pattern and is caused by a deficiency of the lysosomal enzyme, acid β-glucosidase (GCase), also known as glucosylceramidase and glucocerebrosidase, resulting from biallelic, loss-of-function mutations in the glucocerebrosidase gene (*GBA1*) [1,2,3,4]. GCase is responsible for hydrolyzing glucosylceramide into glucose and ceramide; its deficiency leads to the accumulation of glucosylceramide-derived glycosphingolipids in lysosomes (primarily macrophages). Deficiency in GCase activity may be associated with protein misfolding and trafficking or degradation of the enzyme during its formation [1,5].

The disorder manifests with a spectrum of features that overlap between the three classically defined phenotypes according to neurological involvement: Type 1 (non-neuronopathic; GD1, OMIM 230800), Type 2 (acute neuronopathic; GD2, OMIM 230900), and Type 3 (chronic neuronopathic; GD3, OMIM 231000) [1,5,6]. The signs and symptoms of GD may initially be non-specific but can include hepatosplenomegaly, anemia, thrombocytopenia, bone mineral density abnormalities, bone pain, growth delay, and pulmonary involvement [5,7,8,9].

The majority of patients with GD have non-neuronopathic (type 1 disease), which is characterized by hematologic sequelae, potentially disabling skeletal complications, and late-onset neurological complications [5]. The other subtypes of GD cause neuronopathic disease, with early central nervous system involvement: type 2 is associated with death in infancy, while type 3 disease causes a variety of neurological manifestations [5]. While neurologic pathology is prominent in GD2 and GD3, these patients often exhibit systemic features of type 1 disease. Furthermore, the initial signs and symptoms of GD may be non-specific.

Estimates of the incidence and prevalence of GD are sparse and can be inconsistent, even within the same region. Previous incidence rates for GD in the general population have variously been estimated at between 0.39 and 5.80 per 100,000 live births [3], and 1.5 per 100,000 live births [10], with a prevalence ranging from 0.70 to 1.75 [3,10]. A synthesis of global data estimates suggests an overall incidence ranging from 0.45 to 25.0/100,000 live births, comprising 0.45 to 22.9/100,000 live births for GD1 in Europe and North America and 1/36/100,000 live births for GD3 in the Asia-Pacific region [11]. Type-specific prevalence estimates per 100,000 population were 0.26 to 0.63 for GD1, 0.02 to 0.08 for GD2 and GD3 (Europe only), and an overall prevalence estimate for unspecified or overall GD types ranging from 0.11 to 139.0/100,000 inhabitants [11]. The highest prevalence rates are recorded for North America, but prevalence estimates vary considerably between regions and are poorly documented for regions other than North America and Europe, with data for GD2 and GD3 particularly limited. The incidence and prevalence of GD are much higher in individuals of Ashkenazi Jewish descent, with a reported birth incidence of up to 1 in 450 births for GD1 [11].

Carriers of a mutant *GBA1* are also at increased risk of Parkinson’s disease, dementia with Lewy bodies, and multiple myeloma [4,12,13,14]. However, these conditions are not a focus of this paper.

Current pharmacological treatment approaches for GD include enzyme replacement therapies (ERTs) and substrate reduction therapies (SRTs), although there is a lack of specific evidence-based guidelines for the treatment and management of the disease, particularly for asymptomatic individuals or those with minimal symptoms. Some differences in efficacy and safety have been observed for the ERTs, imiglucerase (Cerezyme^®^), produced recombinantly using mammalian cells, velaglucerase alfa (VPRIV^®^), produced using human fibroblasts, and taliglucerase alfa (Elelyso^®^), which is produced using plant cells and is not available in all countries [4,15,16,17,18]. Enzyme replacement therapies aim to reduce the underlying enzyme deficiency of GD and are administered intravenously, whereas the glucosylceramide synthase inhibitor SRTs, miglustat (Zavesca^®^) and the more potent ceramide-analog eliglustat (Cerdelga^®^), reduce substrate synthesis and are administered orally. Miglustat is not currently approved for use in children. New therapeutic approaches to reduce GCase are under investigation, including using small-molecule glucocerebrosidase chaperone agents and gene therapies [16,17].

In addition to ERTs and SRTs, symptomatic treatment of GD may include blood cell transfusions, in the case of severe anemia or bleeding, pain medications or other drugs for bone pain or osteoporosis, and orthopedic surgery for painful, damaged joints [19]. In the era of ERTs and SRTs, bone marrow transplantation and splenectomy are seldom used.

Over 300 mutations of *GBA1* have been identified [2,20], which explains in part the wide range of GD manifestations, and only a limited number of relevant genotype-phenotype associations have been identified. Four pathogenic variants, N370S (now reclassified as p.N409S), L444P, 84GG, and IVS2+1, account for approximately 94% of patients and carriers in Ashkenazi Jewish patients, as well as for approximately half of the mutations seen in non-Jewish white patients [20]. As the symptoms of GD vary widely, even in patients with the same genetic mutation, genotype is not a reliable predictor of disease burden at presentation or for guiding treatment decisions regarding prognosis or likely response to therapy. Although there have been significant advances in newborn screening (NBS), the characterization of phenotypes, and the identification and integration of disease biomarkers into clinical practice, there remains an acute need for GD-specific biomarkers with diagnostic and prognostic potential, as well as for monitoring treatment response and disease progression.

The early diagnosis and timely initiation of appropriate treatment of GD can improve the prognosis of the disease and are essential for alleviating symptoms, reducing the risk of complications, avoiding inappropriate procedures, and improving quality of life [12]. However, the molecular complexity and relative rarity of GD, low awareness among healthcare professionals, the presence of non-specific, complex, or minimal clinical manifestations, and the lack of access to appropriate diagnostic testing often result in delayed diagnosis or misdiagnosis. It is not uncommon for patients to be referred to multiple specialists before a definitive diagnosis is established.

This narrative review examines the current evidence for the role of biomarkers in GD, with a particular focus on glucosylsphingosine (lyso-Gb1).

## 2. Disease Biomarkers in Gaucher Disease

A biomarker can be defined as “a characteristic that is objectively measured and evaluated as an indicator of normal biological processes, pathogenic processes, or pharmacologic responses to a therapeutic intervention” [21]. To describe the role of a biomarker in research and clinical practice and ensure that it is adequate for its intended purpose, it should be validated according to accepted criteria, as defined by the FDA-NIH Biomarker Working Group (https://www.ncbi.nlm.nih.gov/books/NBK326791/ accessed on 21 January 2026). Candidate biomarkers for lysosomal storage diseases such as GD fall into two categories: 1) metabolites or proteins that accumulate in body fluids or tissues as a direct consequence of the primary lysosomal defect or 2) molecules reflecting the effect of the primary lysosomal defect on the function of the involved cell, tissue, or organ [22].

An ideal biomarker has diagnostic utility, provides an indirect assessment of disease activity, and assists in monitoring the effectiveness of therapeutic interventions. Numerous plasma biomarkers have been used to aid in the diagnosing, prognosis, monitoring, and pathophysiologic understanding of GD. These include alkaline phosphatase, tartrate-resistant acid phosphatase (TRAP), angiotensin-converting enzyme (ACE), chemokine ligand 18/pulmonary and activation-regulated chemokine (CCL18/PARC), chitotriosidase, high-density lipoprotein and total cholesterol, macrophage inflammatory proteins 1α and 1β (MIP-1α & MIP-1β or CCL3 & CCL4), glycoprotein nonmetastatic melanoma B (gpNMB, osteoactivin), and ferritin [8,23,24,25,26,27] (Table 1).

Several of these biomarkers are subject to the influence of other factors or are not GD-specific and, therefore, of limited use in diagnosis or monitoring, although they may be useful as first-line screening tests when a diagnosis of GD is suspected. Two examples are the wide use of chitotriosidase and chemokine CCL18 as diagnostic markers in GD. Both biomarkers are elevated in the plasma of patients with GD relative to healthy controls and decrease during disease-specific treatment; however, neither is central to disease pathology or specific to GD [23,24,25]. Elevated chitotriosidase activity is apparent in over 90% of symptomatic patients with GD [28]; however, elevated chitotriosidase levels also occur in other lysosomal storage disorders and inflammatory processes [27,29]. This is also the case with CCL18, as elevated levels are also found in various other diseases, including cancers and chronic inflammatory conditions [30].

An additional problem in the use of chitotriosidase as a biomarker in GD is that approximately 6% of the European population (and even higher percentages in people of Asian ancestry) has no chitotriosidase activity due to homozygosity for duplication of 24 bp in exon 11 of the *CHIT1* gene (dup24, rs3831317), preventing the formation of the active enzyme [31,32]. However, dup24, the main mutation described, is not the only one with implications for the diagnosis and therapeutic monitoring of patients. Some polymorphisms, such as G102S (c.304G>A, p.G102S, rs2297950), G354R (c.1060G>A, p.G354R, rs9943208), and A442V (c.1325C>T, p.A442V, rs1065761), have been associated with reduced chitotriosidase activity [31,33,34].

Research to find more specific and sensitive biomarkers has identified the deacylated form of glucocerebroside, lyso-Gb1 (glucosylsphingosine, also known as lyso-GL1 and GlcSph), as a candidate biomarker for the diagnosis and monitoring of GD [6,30].

## 3. Diagnosis and Prognosis

There is consensus support for universal NBS using dried blood spots (DBSs) for lysosomal storage diseases such as GD, based on evidence that earlier onset and progression of the signs and symptoms of GD correlate with more severe disease and a higher risk of morbidity, and recognizing that diagnosis is commonly delayed for several years after the first clinical and laboratory signs manifest [7]. If individuals with GD who do not require immediate treatment are identified, close monitoring with annual or more frequent assessments should follow. Laboratory testing based on measurement of lysosomal GCase activity in fresh peripheral blood leukocytes is recommended for individuals with signs and symptoms suggestive of GD, especially for those of Ashkenazi Jewish ancestry, followed by definitive diagnosis using whole-*GBA1* sequencing to confirm an initial diagnosis [7]. Kishnani et al. have proposed a useful algorithm for the diagnosis and evaluation of GD [7].

The prognosis of GD is variable and depends on several factors, including the disease type, the age at diagnosis, the timeliness of treatment initiation, and the patient’s overall health [4,5]. With appropriate treatment, patients with non-neuronopathic disease (GD1) can have a near-normal life expectancy, with improvements in hepatosplenomegaly, anemia, and thrombocytopenia, and stable or slowly progressing bone complications, although residual skeletal abnormalities may persist, and late-onset neurological manifestations may develop. The severe and rapidly progressing nature of the rarer GD2 phenotype, which primarily manifests in infants, has a poorer prognosis [4,5]. Children with GD2 may experience neurological decline and severe complications in infancy or early childhood and have significantly reduced life expectancy. Although a milder form than GD2 and with a later onset of symptoms in childhood or adolescence, the prognosis of GD3 varies widely. While some patients may experience a relatively stable disease course, more significant neurological involvement may develop, and GD3 is generally associated with reduced life expectancy in adulthood. The most severe and rarest form of GD, perinatal lethal type GD, is characterized by the earliest onset of symptoms, and most individuals do not survive beyond infancy [4].

Although of limited value in predicting prognosis or guiding treatment decisions, certain genotype–phenotype correlations for *GBA1* variants have been established, despite significant overlap in clinical manifestations between GD phenotypes [9,20,23,35].

## 4. Lyso-Gb1

### 4.1. Pathophysiological Role

Raghavan et al. first isolated and characterized lyso-Gb1 as a natural constituent of the spleens of two patients with GD in the 1970s [36]. Elevated levels of lyso-Gb1 were subsequently reported in the gray matter of the brain and cerebellum of neuronopathic (type 2 and 3) GD patients, suggesting a potential neurotoxic role, and lyso-Gb1 was also detected in other organs, including the spleen and liver, in patients with type 1, 2, and 3 GD [37,38]. Elevated levels of lyso-Gb1 are also found in the red blood cells (RBCs) of untreated patients with GD, compared with healthy controls [39], which may help explain anemia and ischemic events, although the clinical implications in GD are unclear. However, ERT has been shown to decrease levels of lyso-Gb1 in RBCs [40].

Lyso-Gb1 is a direct metabolite of glucosylceramide and is produced by a process of deacetylation mediated by acid ceramidase activity [27,41]. As a result of GCase deficiency, glucosylceramide progressively accumulates in lysosomal macrophages in the reticuloendothelial system, forming pathologic Gaucher cells. Accumulation of lyso-Gb1 in Gaucher cells has a clear role in the disease-related pathology of GD. Plasma lyso-Gb1 levels are between 200- and 500-fold higher in patients with GD compared to healthy controls [6,40,42], and an excess of lyso-Gb1 is understood to induce hepatosplenomegaly and pancytopenia and contribute to the impairment of osteoblasts, leading to the bone disease commonly experienced by patients with GD1.

As noted, carriers of a mutant *GBA1* are at increased risk of other conditions, including Parkinson’s disease and multiple myeloma. Although these conditions are not a focus of this paper, it has been suggested that lyso-Gb1 promotes the pathological accumulation of a disease-specific biomarker for PD, α-synuclein, particularly in patients with GD3 and carriers of the L444P variant, including age-dependent increases in carriers [43], increasing the risk of Parkinson’s disease in patients with GD and carriers [44]. This suggests a potential role for acid ceramidase and *GBA2* as therapeutic targets for preventing and treating *GBA*-associated Parkinson’s disease [44]. Further implications of the role of α-synuclein aggregation and toxicity in GD, where *GBA1* mutations are involved, are unclear but suggest an interplay between GD and Parkinson’s disease. A summary of the in vitro and in vivo evidence for the role of lyso-Gb1 as a pathogenic mediator in GD has been reported in a systematic literature review by Revel-Vik et al. [24]. Mistry and colleagues, using a murine model of GD in which the *GBA1* gene was deleted, demonstrated widespread dysfunction in macrophages, osteoblasts, and other cell types, causing severe osteoporosis arising from defective osteoblastic bone formation due to the inhibitory effect on protein kinase C of accumulating lyso-Gb1 and glucosylceramide in osteoblasts [45]. Lyso-Gb1 has since been implicated in mediating several key features of GD1 related to immune dysregulation and skeletal disease [46,47,48,49]. Lyso-Gb1 may also act as an antigen driving B-cell and plasma cell activation. In a murine model of GD, which was associated with increased lyso-Gb1 levels and, in most cases, monoclonal gammopathy, eliglustat treatment reduced lymphoproliferation [50], suggesting that glycosphingolipids could stimulate the proliferation of mature B lymphocytes and plasma cells. Moreover, Lyso-Gb1 has been shown to influence antigen-specific type II natural killer T-cells, which lead to immune dysfunction in the pathogenesis of monoclonal B-cells in GD-associated myeloma [49], while monoclonal immunoglobulins from patients with monoclonal gammopathy in GD were specific against lyso-Gb1, which mediated the activation of B lymphocytes and plasma cells [49,51].

An accumulation of lyso-Gb1 may already be present prenatally, with clinical manifestations in affected individuals ranging from lethal neonatal complications to asymptomatic presentation [16]. Therefore, lyso-Gb1 has an early diagnostic and pathophysiological role with utility in the prenatal or perinatal diagnosis of GD and in NBS. New insights into the pathophysiological behavior of lyso-Gb1 at the fetal–neonatal level, obtained by analyzing the data of three newborns of GD-affected mothers, suggest that transplacental transfer of lyso-Gb1 is the most likely route of transmission from mother to fetus [52]. However, caution is advised in interpreting neonatal lyso-Gb1 (i.e., maternal contribution vs. infant metabolism) until the implications of placental transfer mechanisms for enhancing newborn screening programs are better understood.

A large study of approximately 250,000 neonates screened over eight years for lysosomal storage disorders in northeast Italy demonstrated that lyso-Gb1 measured by liquid chromatography-tandem mass spectrometry (LC-MS/MS) assay in DBS is predictive of GD severity [53]. Increased levels of lyso-Gb1 in DBS had a 100% positive predictive value and correlated with disease burden, consistent with the genotype; significantly higher plasma concentrations of lyso-Gb1 were measured in patients harboring neuronopathic alleles than in patients with non-neuronopathic GD. Levels were 13 to 56 times higher in patients with GD2 than those in GD1 patients, suggesting that early and significant accumulation of lysoGb1 may be predictive of clinical phenotype in GD. The decay of lyso-Gb1 after ERT appears to follow an exponential curve, with lyso-Gb1 levels half-normalized around 15 months, followed by a much slower change in spleen volume [54]. Values of lyso-Gb1 plateau at around 8 years, although there is considerable inter- and intra-individual variability and non-linear behavior related to disease burden and ERT dose [55,56].

Although levels of lyso-Gb1 may also vary over time and depending on age [57,58], other studies have also demonstrated the utility of lyso-Gb1 as a predictor of genotype/phenotype and disease severity [6,8,42,58,59]. Conversely, GCase activity does not appear to correlate with GD subtype or severity [53].

Despite levels of inter- and intra-individual variability that have been observed in some studies, lyso-Gb1 concentrations have been shown to correlate with baseline disease burden, including moderate-to-strong correlations between plasma lyso-Gb1 and spleen and liver volumes and hemoglobin level [55,60,61]. Moderate and highly statistically significant correlations with disease severity, as measured by the Disease Severity Scoring System (GD-DS3), have been demonstrated in patients with type 1 or unclassified GD, reinforcing the application of lyso-Gb1 as a predictive biomarker for phenotypic severity [23]. As early accumulation of lyso-Gb1 may be suggestive of disease progression in children with type 1 GD, determination of lyso-Gb1 levels may support a decision to initiate treatment in patients otherwise without, or with only mild, signs or symptoms of the disease [62].

Table 2 summarizes the studies discussed that report on the role of lyso-Gb1 as a biomarker in GD.

### 4.2. Diagnostic Role

Plasma and tissue levels of lyso-Gb1 in healthy individuals are undetectable or found only at trace levels, whereas significantly higher levels are detectable in individuals with non-neuronopathic and neuronopathic GD, and in neuronopathic GD versus non-neuronopathic GD [53,59], suggesting a role for screening for lyso-Gb1 as a diagnostic tool. Indeed, lyso-Gb1 has become accepted as a statistically reliable diagnostic and pharmacodynamic biomarker for GD, able to identify pathological levels in GD patients that are not present in healthy controls, GD carriers, or patients with other lysosomal storage disorders, and is the most specific and sensitive biomarker for diagnosing GD, regardless of GD phenotype [23,29,30,68,69]. Lyso-Gb1 has an early diagnostic and pathophysiological role in pre-symptomatic prenatal testing or neonatal (newborn) screening (NBS) [7,17,24,53,58,69].

The use of LC/MS/MS allows levels of lyso-Gb1 to be reliably quantified in plasma, providing diagnostic information and reflecting patients’ response to therapy [6,24,25,39,60,66,69]. Furthermore, lyso-Gb1 is also readily accessible and quantifiable in DBSs [24,59,63,65,69,70]: analyzing lyso-Gb1 levels and GCAse enzyme activity in DBS, followed by genetic confirmation, offers advantages over plasma analysis, including requiring a smaller volume of blood, straightforward collection of samples that are stable at room temperature, and ease of transport between laboratory facilities [8,23,24,63]. There are some limitations to measuring lyso-Gb1 in DBS, including the necessity for highly specific and sensitive assay methods, and possible issues with sample integrity and variations in hematocrit level, which can influence results.

Currently, several different plasma cut-offs and assay contexts have been described. Two study groups that investigated the use of lyso-Gb1 as a biomarker for GD in patients with GD, obligate carriers, and healthy controls detected marked increases in levels of lyso-Gb1 (>200-fold) in patients with symptomatic GD1 compared with healthy controls [6], and identified 12 ng/mL as an ideal cut-off level to differentiate GD from healthy controls, and between patients with GD and GD carriers [30]. In another prospective study that defined lyso-Gb1 as a key biomarker of GD at diagnosis, Murugesan et al. found that a 4 ng/mL cut-off distinguished patients with GD from healthy controls with 100% sensitivity and specificity [42], which is similar to the 5.4 ng/mL identified by Irún et al. [64]. However, current variations in the methodologies and units of measurements used by laboratories to analyze lyso-Gb1 mean there is not yet a consensus on a reliable cut-off level to differentiate patients with GD from carriers and healthy controls.

Nevertheless, lyso-Gb1 can still be considered a valuable diagnostic aid and is, in addition, a reliable biomarker for baseline disease burden with utility for monitoring disease and therapeutic response [24,30,59,63,64,69].

### 4.3. Role in Treatment Initiation, Response Evaluation, and Monitoring

Measuring lyso-Gb1 may provide an accurate reflection of disease burden and an early indicator of disease progression, supporting decisions on when to initiate treatment in patients with GD. A study by Dinur et al. found that all patients with GD had lyso-Gb1 levels greater than 9 ng/mL [8], but that higher levels (>250 ng/mL) were predictive of initiating therapy [67]. Although they cautioned that variable methodologies for analyzing lyso-Gb1 and a lack of uniformity in the units for reporting lyso-Gb1 currently prevent the adoption of specific cut-off values in clinical practice, a significant elevation from diagnostic levels of lyso-Gb1 indicates more severe disease and may be used to guide treatment decisions [67].

Lyso-Gb1 has been shown to more directly reflect the disease course of GD than other currently investigated biomarkers and has been shown to correlate with response to therapy in patients of all ages [30,54,58,59,60,63,64,66,67,69,71]. Mean lyso-Gb1 levels decrease to approximately half of their initial value within one year of initiating ERT, although with considerable heterogeneity in individual patient responses [42]. Lyso-Gb1 levels then stabilized within 3 to 4 years of ongoing ERT, although there was a residual elevation at 13.5 times greater than the reference range at five years of treatment [42], which was also dependent on treatment.

Kleytman et al. further demonstrated the value of lyso-Gb1 for monitoring treatment response in a real-world study of patients with GD1 [71]. Switching patients stabilized on long-term ERT for a minimum of two years to an SRT (eliglustat) incrementally reversed GD activity indicators, nearing healthy control levels in some patients [71]. There were significant improvements in spleen volume and platelet counts without deterioration of liver function, accompanied by a reduction in the three plasma biomarkers of GD activity, lyso-Gb1, chitotriosidase, and gpNMB [71]. Furthermore, long-term data from phase 2 extension and phase 3 SRT programs show sustained declines in lyso-Gb1 levels over years accompanied by decreases in spleen and increases in liver volumes and hemoglobin and platelet levels [60,72,73], reinforcing the value of lyso-Gb1 for monitoring therapeutic response. Of note, in a real-world study of 38 patients switched from long-term ERT to eliglustat [71], there was a further reduction in lyso-Gb1, chitotriosidase, and gpNMB, while in a small cohort-matched study [74], there was a trend toward greater reductions in biomarker levels in patients switched from ERT to SRT.

Levels of lyso-Gb1 also correlate well with disease burden [30,42,60,63] and with other indicators of disease severity, including chitotriosidase, CCL18, spleen volume, and liver volume [42]. There is a rapid and significant reduction in lyso-Gb1 after ERT infusion, with overall stability despite substantial year-to-year ‘noise’ in biomarker level [57]. However, the individual variability in lyso-Gb1 levels [57,59,63] and current variations in the methodologies and units of measurement used by laboratories for the analysis of lyso-Gb1 mean that there is not yet a consensus on a reliable cut-off to assign levels for diagnosis, treatment initiation, and response to therapy. Additional research is required to determine the appropriate levels for use in clinical practice. To this end, the International Working Group for Gaucher Disease (IWGGD) Laboratory Working Group is undertaking research toward standardizing the methodology for measuring lyso-Gb1 (https://iwggd.com/iwggd-working-group-on-gaucher-disease/ accessed on 19 March 2025). Reflecting the pharmacodynamic behavior of lyso-Gb1, a goal of the IWGGD is to introduce standardized sample timing (e.g., consistent plasma trough collection methodologies) to ensure accurate diagnosis and improve practical guidance for DBS monitoring [25]. The influence of the matrix (plasma vs. DBS) utilized for GD testing, the testing platform, and calibration standards on determining threshold values further emphasizes the importance of the call for further efforts towards standardization [25].

Nevertheless, measurement of lyso-Gb1 already provides valuable information to facilitate decision-making on whether a therapy is, or is not, achieving the expected outcomes, to monitor treatment adherence, and to assist clinicians in optimizing individual dosing regimens based on the patient’s disease burden. Finally, in addition to having a role in decisions to initiate ERT or SRT, the correlation between lyso-Gb1 levels and disease status may have clinical utility for optimizing individual treatments [6]. Serial and periodic monitoring of lyso-Gb1 may thus facilitate the appropriate allocation of healthcare resources while ensuring the optimal management of patients with GD.

### 4.4. Analysis and Reporting of Lyso-Gb1

As noted, there is currently no consensus on definitive laboratory cut-off values for lyso-Gb1 levels that differentiate patients with GD from carriers and healthy controls, or on appropriate levels for diagnosis, disease severity, and response to therapy. Collaboration and cross-validation between laboratories are necessary to ensure comparability of inter-laboratory biomarker data [24] but, currently, there is a lack of standardization in the reporting of lyso-Gb1 levels, which may be expressed in ng/mL or in SI and ERNDIM units (nmol/L).

While work is ongoing to define the ideal lyso-Gb1 cut-off points of relevance to GD, the recently published American College of Medical Genetics and Genomics (ACMG) Technical Standard for biomarker testing for lysosomal diseases is a valuable resource for clinical laboratory geneticists to assist in providing consistent, accurate, and standardized quality clinical laboratory genetic services [22]. The ACMG guidelines provide clear and detailed information on the methodology for analyzing and reporting lyso-Gb1 and other biomarkers in lysosomal storage diseases. We refer you to the ACMG Technical Standard [22] for guidelines on clinical indications for testing, sample type, collection, handling, and storage, validation of methods and reference ranges, calibration and quantitation, proficiency testing, staff training, and competency assessments, as well as specific methods of analytical methods for biomarker analysis, interpretation, and result reporting.

## 5. Discussion

As this narrative review reinforces, the current evidence supports the role of lyso-Gb1 as a reliable, specific, and sensitive biomarker in GD, surpassing other markers such as chitotriosidase, alkaline phosphatase, TRAP, ACE, and CCL18/PARC. Lyso-Gb1 has become an important biomarker in the disease, facilitating valuable insights into disease severity, progression, and treatment response. There is compelling evidence for the value of lyso-Gb1 as a sound indicator of substrate accumulation resulting from GCase deficiency and its role in the pathogenesis of GD, with a corresponding role as a pathogenic mediator of disease.

Lyso-Gb1 has a broad range of utility in GD, from NBS and assessing baseline disease burden to the diagnosis and prediction of disease severity, guiding treatment decisions, monitoring treatment effectiveness, and providing insight into disease progression. The evidence supports lyso-Gb1 as a crucial biomarker for early detection of treatment issues, even before clinical symptoms appear.

Due to the relative rarity of GD and complex and often non-specific or minimal clinical manifestations, there is low awareness of GD among healthcare professionals. This is compounded by limited access to appropriate standardized diagnostic testing facilities that contribute to delayed diagnosis or misdiagnosis. This is particularly important, as early diagnosis and timely, appropriate therapeutic intervention can improve the prognosis of GD while relieving symptoms, reducing complications, and improving quality of life. As a component of NBS programs, lyso-Gb1 screening assays could reduce diagnostic delay and trigger the early initiation of appropriate GD-specific therapy.

Looking to the future, the metabolic biomarker lyso-Gb1 may be useful to assess the efficacy of novel therapeutic agents.

## 6. Materials and Methods

### Data Sources and Search Strategy

A literature search to identify relevant articles was conducted in PubMed and Embase using the following search terms: (“Gaucher disease” [MeSH Terms] OR (“Gaucher” [All Fields] AND “disease” [All Fields]) OR “Gaucher disease” [All Fields]) AND (“sphingosyl beta glucoside” [Supplementary Concept] OR “sphingosyl beta glucoside” [All Fields] OR “glucosylsphingosine” [All Fields]); (“Gaucher disease” [MeSH Terms] OR (“Gaucher” [All Fields] AND “disease” [All Fields]) OR “Gaucher disease” [All Fields]) AND (“lyso” [All Fields] AND “GL1” [All Fields]) (PubMed) and (‘Gaucher disease’/exp OR ‘Gaucher disease’) AND (‘glucosylsphingosine’/exp OR glucosylsphingosine); (‘Gaucher disease’/exp OR ‘Gaucher disease’) AND (‘lyso GL1’ OR (lyso AND GL1)) (Embase). The reference sections of identified articles were manually screened to identify additional pertinent studies. English-language studies were included in this review if they reported the evaluation of biomarkers in Gaucher disease, with sensitivity and specificity outcomes of interest for Gaucher disease; the role of biomarkers in the diagnosis, treatment, monitoring, or prognosis of Gaucher disease. Reviews and meta-analyses were used to identify additional relevant studies but were otherwise excluded. However, pooled analyses were included. Due to the heterogeneity of the available studies, the results of this review are summarized narratively.

## 7. Conclusions

Diagnostic cut-off levels of lyso-Gb1 are becoming more evident, but further work is needed to describe the cut-offs that establish the safe range for disease severity and treatment decisions. Ongoing research will enhance our understanding of its role as a pathogenic and prognostic factor in GD and how treatment-related declines in lyso-Gb1 concentrations correlate with validated disease severity scores to determine optimal metabolic responses to therapy.

In conclusion, the literature supports the utility of lyso-Gb1 as a sensitive biomarker for GD diagnosis and for predicting the clinical course and guiding the personalized management of patients with GD as it is a direct measure of substrate accumulation that reflects therapeutic response.

## Figures and Tables

**Table 1:** Disease and diagnostic biomarkers in Gaucher disease (GD).

Biomarker ^a^	Diagnostic Utility	Monitoring Utility	Limitations
Chemokine ligand 18/pulmonary and activation-regulated chemokine (CCL18/PARC)	Can aid in diagnosis; levels significantly elevated in GD	Used for routine follow-up, especially in ChT-deficient patients	Lacks disease specificity and can be elevated in other chronic inflammatory conditions or cancers.
Chitotriosidase (ChT)	Useful for initial screening and a strong indicator of macrophage activity; directly proportional to Gaucher cell burden	Widely used for monitoring disease activity and treatment follow-up	Not specific to GD. Approximately 6% of the population has a genetic deficiency resulting in undetectable levels, leading to false negatives.
Glucosylsphingosine (Lyso-Gb1)	High sensitivity and specificity; significantly elevated in GD patients and reliably quantified in plasma and DBS	Considered a valuable diagnostic aid and reliable biomarker for monitoring disease burden, progression and treatment response	Requires specific and specialized testing; possible issues with sample integrity and hematocrit level variations in DBS. Lack of consensus on reliable cut-off levels.
Macrophage inflammatory proteins 1α and 1β (MIP-1α & MIP-1β)	Elevated levels in patients with symptomatic GD, but some overlap with healthy individuals	Used primarily as a monitoring biomarker for assessing skeletal disease and response to ERT	Limited disease specificity for diagnostic purposes; high levels of MIP-1β persisting during therapy associated with ongoing skeletal disease.
Tartrate-resistant acid phosphatase (TRAP)	Generally elevated at diagnosis of GD	Levels tend to decline during ERT; specific to bone resorption	Not highly specific to GD; can be elevated in other conditions.

^a^ Other biomarkers of limited specificity and utility in Gaucher disease include Alkaline phosphatase, Angiotensin-converting enzyme (ACE), Glycoprotein nonmetastatic melanoma B (gpNMB), Platelet count and hemoglobin concentration. ERT, enzyme replacement therapy.

**Table 2:** The utility of lyso-Gb1 as a specific biomarker for early detection, predicting clinical course, and guiding treatment decisions in Gaucher disease (GD).

References	Study Design	Population	Key Findings
Dekker et al., 2011 [6]	P, M	64 GD1 34 GD carriers 28 healthy controls	Mean plasma lyso-Gb1 increased >200-fold in GD1 pts but not in healthy controls or obligate carriers. Lyso-Gb1 significantly correlated with baseline disease severity and biomarkers ChT and CCL18, but not MIP-1β.
Rolfs et al., 2013 [30]	R, S	98 GD pts 13 GD carriers 148 healthy controls 262 pts with other LSD	Levels of plasma lyso-Gb1 > 12 ng/mL identified in GD pts but not in healthy controls, GD carriers, and pts with other LSD. Lyso-Gb1 was more sensitive and specific than ChT and CCL18 at diagnosis based on a 12 ng/mL cut-off, which was established with an ideal sensitivity and specificity of 100% in 521 analyzed samples.
Murugesan et al., 2016 [42]	P	169 GD1 41 healthy controls	Plasma lyso-Gb1 levels increased by >200-fold in untreated pts with GD1 vs. healthy controls. Pts with GD1 and healthy controls were distinguished by a cut-off of 4 ng/mL, both with a sensitivity and specificity of 100%. Plasma lyso-Gb1 values between pts with GD1 and healthy controls did not overlap.
Chipeaux et al., 2017 [39]	P, M	15 GD1 11 healthy controls	Lyso-Gb1 levels in plasma and RBCs 1 or 2 orders of magnitude higher in pts with GD1 vs. healthy controls.
Arkadir et al., 2018 [54]	R	25 GD pts	Lyso-Gb1 levels decreased following ERT and platelet counts and hemoglobin increased and spleen volume clearly decreased. Lyso-Gb1 shown to be a reliable biomarker for monitoring response to ERT.
Hurvitz et al., 2019 [59]	R	35 mild GD1 34 severe GD1 12 type 3 GD	Significantly higher levels of lyso-Gb1 in DBS associated with more symptomatic disease at baseline in children who subsequently underwent ERTs, and in severe vs. mild disease.
Cozma et al., 2020 [63]	R	19 ERT-treated GD pts	Lyso-Gb1 reliably detected in DBS samples over a 3-year period. Following an involuntary treatment break, the separation of lyso-Gb1 levels “under treatment” versus “not under treatment” was identified with high sensitivity and specificity.
Irún et al., 2020 [64]	R	47 GD pts 19 GD carriers 42 healthy controls 37 pts with other LSD	Pts with GD pts, but not carriers or controls, had plasma lyso-Gb1 levels >5.4 ng/mL at diagnosis. Lyso-Gb1 levels significantly correlated with ChT activity and CCL18/PARC, but not with disease burden.
Saville et al., 2020 [65]	R	23 GD pts 12 non-neuronopathic, 11 neuronopathic 156 controls 3 GD carriers 37 other-IMD	Median lyso-Gb1 concentrations were detected in DBS from neuronopathic GD pts vs. non-neuronopathic GD pts and all GD pts were higher vs. controls.
Dinur et al., 2021 [55]	LO	135 ERT-treated GD1 pts	Lyso-Gb1 in DBS shown to be a reliable biomarker for monitoring ERT-treated pts with GD1.
Dinur et al., 2022 [8]	CS	99 GD pts	Median (range) lyso-Gb1 levels in DBS were lower in pts with GD1 and mild *GBA1* variants, vs. GD1 and severe variants and neuronopathic GD, whereas pts with heterozygous *GBA1* variants had higher lyso-Gb1 levels vs. wild-type *GBA1*.
Ida et al., 2022 [66]	O, M	20 ERT-treated GD pts 8 GD1, 9 GD2, 3 GD3	Plasma lyso-Gb1 levels were non-significantly lower in pts who achieved all therapeutic goals vs. those without 100% achievement. No significant differences in lyso-Gb1 levels by GD type or genotype.
Curado et al., 2023 [23]	P, M	160 naïve pts 114 GD1 46 unclassified	Lyso-Gb1 values at enrollment had a moderate and statistically highly significant correlation with disease severity in all GD pts.
Dinur et al., 2023 [67]	R	97 naïve GD pts 87 GD1, 10 neuronopathic	A cut-off of lyso-Gb1 >250 ng/mL in DBSs was predictive for treatment initiation (sensitivity 71%, specificity 87.5%). A significant elevation from diagnostic levels indicative of more severe disease and may have utility for guiding treatment decisions.
Peterschmitt et al., 2023 [60]	P	66 naïve GD1 pts	Moderate-to-strong correlations between plasma lyso-Gb1 levels and baseline disease burden, which improved with ERT treatment in 26 pts from a phase 2 open-label study and 40 pts from a placebo-controlled phase 3 study with ≤8 and ≤4.5 years of follow-up, respectively.
Dubiela et al., 2024 [57]	P	75 GD pts 56 GD1, 19 GD3	Analysis of 8 years of data showed higher variability of lyso-Gb1 levels in GD1 than GD3. ERT rapidly and significantly reduced lyso-Gb1 levels.

CCL18, chemokine ligand 18; CCL18/PARC, CCL18/pulmonary and activation-regulated chemokine; ChT, chitotriosidase; CS, cross-sectional; DBS, dry blood spot; ERT, enzyme replacement therapy; GD, Gaucher disease; GD1, type 1 GD; GD2, type 2 GD; GD3, type 3 GD; IMD, inherited metabolic disorder; LO, longitudinal observational; Lyso-Gb1, glucosylsphingosine; LSD, lysosomal storage disorder; M, multicenter; MIP, macrophage inflammatory protein; O, observational; P, prospective; pts, patients; R, Retrospective; RBC, red blood cell; vs., versus.

## Data Availability

No new data were created or analyzed in this study.

## Abbreviations

The following abbreviations are used in this manuscript:

ACE	Angiotensin-converting enzyme
CCL18	Chemokine ligand 18
DBS	Dried blood spots
ERT	Enzyme replacement therapy
*GBA1*	Glucocerebrosidase gene
GCase	Acid β-glucosidase
GD	Gaucher disease
GD1	Non-neuronopathic Gaucher disease
GD2	Acute neuronopathic Gaucher disease
GD3	Chronic neuronopathic Gaucher disease
gpNMB	Glycoprotein nonmetastatic melanoma B
LC-MS/MS	Liquid chromatography-tandem mass spectrometry
Lyso-Gb1	Glucosylsphingosine (also known as lyso-GL1 and GlcSph)
MIP	Macrophage inflammatory protein
NBS	Newborn screening
PARC	Pulmonary and activation-regulated chemokine
SRT	Substrate reduction therapy
TRAP	Tartrate-resistant acid phosphatase
